# Off-the-Shelf Cord-Blood Mesenchymal Stromal Cells: Production, Quality Control, and Clinical Use

**DOI:** 10.3390/cells13121066

**Published:** 2024-06-19

**Authors:** Tiziana Montemurro, Cristiana Lavazza, Elisa Montelatici, Silvia Budelli, Salvatore La Rosa, Mario Barilani, Cecilia Mei, Paolo Manzini, Ilaria Ratti, Silvia Cimoni, Manuela Brasca, Daniele Prati, Giorgia Saporiti, Giuseppe Astori, Francesca Elice, Rosaria Giordano, Lorenza Lazzari

**Affiliations:** 1 Unit of Cellular and Gene Therapy, Fondazione IRCCS Ca’ Granda Ospedale Maggiore Policlinico, 20122 Milano, Italy; tiziana.montemurro@policlinico.mi.it (T.M.); cristiana.lavazza@policlinico.mi.it (C.L.); elisa.montelatici@policlinico.mi.it (E.M.); silvia.budelli@policlinico.mi.it (S.B.); salvatore.larosa@policlinico.mi.it (S.L.R.); m.barilani@gmail.com (M.B.); ceciliamei1992@gmail.com (C.M.); paolo.manzini@policlinico.mi.it (P.M.); lorenza.lazzari@policlinico.mi.it (L.L.); 2 Milano Cord Blood Bank and Center of Transfusion Medicine, Fondazione IRCCS Ca’ Granda Ospedale Maggiore Policlinico, 20122 Milano, Italy; ilaria.ratti@policlinico.mi.it (I.R.); silvia.cimoni@policlinico.mi.it (S.C.); manuela.brasca@policlinico.mi.it (M.B.); daniele.prati@policlinico.mi.it (D.P.); 3 Bone Marrow Transplantation and Cellular Therapy Center, Hematology Unit, Fondazione IRCCS Ca’ Granda Ospedale Maggiore Policlinico, 20122 Milano, Italy; giorgia.saporiti@policlinico.mi.it; 4 Laboratory of Advanced Cellular Therapies and Haematology Unit, San Bortolo Hospital, AULSS8 “Berica”, 36100 Vicenza, Italy; giuseppe.astori@aulss8.veneto.it (G.A.); francesca.elica@aulss8.veneto.it (F.E.)

**Keywords:** umbilical cord blood, mesenchymal stromal cells, cell therapy, innovative therapies, transplantation

## Abstract

Background Recently, mesenchymal stromal cells (MSCs) have gained recognition for their clinical utility in transplantation to induce tolerance and to improve/replace pharmacological immunosuppression. Cord blood (CB)-derived MSCs are particularly attractive for their immunological naivety and peculiar anti-inflammatory and anti-apoptotic properties. Objectives: The objective of this study was to obtain an inventory of CB MSCs able to support large-scale advanced therapy medicinal product (ATMP)-based clinical trials. Study design: We isolated MSCs by plastic adherence in a GMP-compliant culture system. We established a well-characterized master cell bank and expanded a working cell bank to generate batches of finished MSC(CB) products certified for clinical use. The MSC(CB) produced by our facility was used in approved clinical trials or for therapeutic use, following single-patient authorization as an immune-suppressant agent. Results: We show the feasibility of a well-defined MSC manufacturing process and describe the main indications for which the MSCs were employed. We delve into a regulatory framework governing advanced therapy medicinal products (ATMPs), emphasizing the need of stringent quality control and safety assessments. From March 2012 to June 2023, 263 of our Good Manufacturing Practice (GMP)-certified MSC(CB) preparations were administered as ATMPs in 40 subjects affected by Graft-vs.-Host Disease, nephrotic syndrome, or bronco-pulmonary dysplasia of the newborn. There was no infusion-related adverse event. No patient experienced any grade toxicity. Encouraging preliminary outcome results were reported. Clinical response was registered in the majority of patients treated under therapeutic use authorization. Conclusions: Our 10 years of experience with MSC(CB) described here provides valuable insights into the use of this innovative cell product in immune-mediated diseases.

## 1. Introduction

Mesenchymal stromal cells (MSCs) are multipotent non-hematopoietic adult cells that were initially discovered in the bone marrow (BM) [1] and since have been identified in adipose tissue [2], amniotic fluid [3], dental pulp [4], placenta [5], umbilical cord blood (CB) [6], and Wharton’s jelly [7]. These MSCs sense and respond to signals from inflamed and injured tissue by secreting extracellular vesicles that contain trophic and immunomodulatory factors [8].

Cord blood is an ethically non-controversial and safe source of transplantable cells [9], carries a low risk for transmission of viral infections, and is easier to collect than BM. CB provides stem and progenitor cells to reconstitute the hematopoietic system, restore immunological functions, and induce or solicit tolerance in solid organ transplantation [10,11,12]. To date > 35,000 transplants have been performed, and > 800,000 CB units are available worldwide for transplantation [13,14]. Since 2000, MSCs have been isolated from full-term CB and found to meet all requirements for the MSC definition [6,15]; they have a typical fibroblast-like morphology, plastic-adherence capability, clonogenic activity, morphological heterogeneity, differentiation ability, and mesenchymal marker expression (CD105, CD73, and CD90), and they do not express CD45, CD34, CD14, CD11b, CD79α, CD19, and HLA-DR surface molecules. However, MSCs derived from perinatal and adult tissues do have some differences in molecular profile, tri-differentiation potentials, proliferation/clonogenic capacities, immunomodulatory functions, and hematopoietic support [16,17,18,19]. Heterogeneity can also arise from multiple cell-type populations within a single MSC source; CB may give rise to both long-living (LL) and short-living (SL) MSC populations with marked differences in lifespan, maximum population doublings, and telomere length at early passages (P0-1), but not at late passages (P5) [20,21]. We previously identified MSC(CB) LL as the best cell type for a GMP large-scale production of off-the-shelf advanced therapy medicinal products (ATMPs). Here, we describe our Good Manufacturing Practice (GMP)-compliant production process for MSC(CB) for clinical applications; we hope to generate interest in the tremendous potential of this innovative therapeutic tool.

## 2. Materials and Methods 

### 2.1. Tissue Procurement

CB was collected in a multiple system bag (Macopharma, Tourcoing, France) with 29 mL of citrate phosphate dextrose after written informed consent from the mother. All procedures (including transportation) complied with international standards [22]. Donor selection complied with national guidelines [23]. Eligible donors were negative for HBsAg, anti-HCV, anti-HIV 1/2, TPHA/VDRL, HIV RNA, HCV RNA, and HBV DNA, as well as any markers required by national rules for epidemic emergencies. The starting material specifications for CB are listed in Supplementary Table S1. We used only CB units that were ineligible for banking (volume < 60 mL or cell count < 1.5 × 10^9^ total nucleated cells).

### 2.2. GMP Facility

In Italy, ATMP production sites must be authorized by the Italian Drug Agency (AIFA) in accordance with GMP guidelines. Therefore, in 2007, the Cell Factory of the Fondazione IRCSS Ca’ Granda Ospedale Maggiore Policlinico in Milan was set up and GMP-certified by AIFA; the facility has maintained its authorization continuously (inspected every 2–4 years). Production and quality-control areas are restricted-access and include Class B-GMP laboratories with class-A biosafety cabinets and changing rooms with air-purification protocols, attire, and accessories appropriate for clean rooms. Personnel are granted access to production areas following training in materials and reagents preparation and other appropriate standard operating procedures to minimize contamination [24]. Manufacturing was monitored continuously for non-viable particulates and for microbial control of air, surfaces, and operators; out-of-specification and out-of-trend incidents were investigated and corrected.

### 2.3. MSC(CB) Manufacturing Process

Reagents for cell-based therapeutics must be GMP grade and comply with European and national regulations; reagent quality directly impacts the quality, safety, and efficacy of the medicinal product. Reagents for MSC manufacturing meet predefined standards and undergo mandated certified analytical testing. The fetal bovine serum (FBS) chosen complies with rules governing TSE transmission in bovine products and all relevant oversight regarding reagents of animal origin in medicinal product manufacturing [25,26].

**Figure 1:**
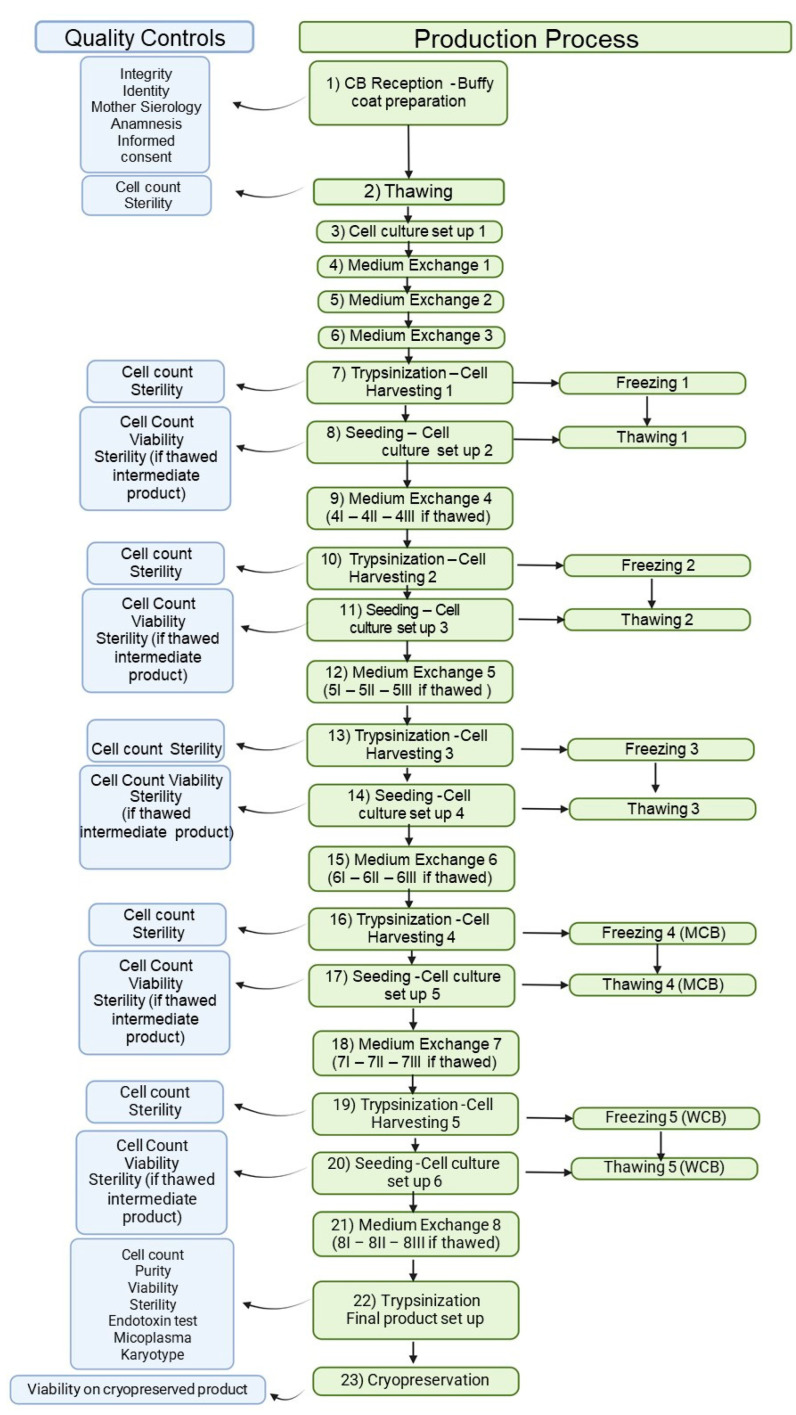
Cord blood-derived mesenchymal stromal cell manufacturing process, with in-process quality controls.

We established a clinical cell bank using modifications of the published pre-clinical protocol (Figure 1) [21]. Briefly, whole blood was centrifuged (800× *g* for 15 min), and the plasma was discarded; mononuclear cells from the buffy coat were seeded at 50–100 × 10^3^ cells/cm^2^ in vented cell culture flasks of alphaMEM complete medium (Macopharma) with 20% qualified FBS (ThermoFisher, Waltham, MA, USA). Cultures were incubated in a humidified atmosphere (37 °C, 5% CO_2_) for 10 d, with culture media changes every 48–72 h. On day +10, cells were harvested by trypsinization (TrypLE Select, ThermoFisher) and subcultured at 1000–4000 cells/cm^2^. Media was changed on days +13 and +20; adherent cells were harvested and re-seeded (1000–4000 cells/cm^2^) on days +17 and +24, with some harvested cells saved in a cryopreservation buffer of 80% normal saline (B. Braun, Melsungen, Germany), 10% dimethyl sulfoxide (DMSO, CRYOSERV, Mylan Institutional, Inc., Canonsburg, PA 15317, USA), and 10% human serum albumin (HSA, Kedrion, Lucca, Italy). This sequence (seeding—medium change—trypsinization—cryopreservation) was repeated through passage 4, thus providing cryopreserved samples for the master cell bank (MCB) from passage 3 (P3), the working cell bank (WCB) from passage 4 (P4), and final product manufacturing following passage of the WBC to passage 5 (P5). The fold expansion (FE) was calculated at each passage (cells harvested/cells seeded), and the MSC production yield was calculated as follows:

theoretical WCB = MCB total × P3 → P4 FE

final product yield = theoretical WCB × P4 → P5 FE

Mean FEs were calculated for P3–P4 (n = 5) and P4–P5 (n = 21), starting from three different batches of CB starting material.

The final product was obtained by an additional 7–10 d of culture. The final product was packaged in a bag (CryoMACS Freezing bag 50, Miltenyi Biotec B.V. & Co., Bergisch Gladbach, Germany) in 20 mL of cryopreservation buffer and frozen at a controlled rate (Nicool Plus, Air Liquide, 75 quai d’Orsay 75321, Paris cedex 07, France or Planer Cryo 560-16, Planer LTD., Sunbury-on-Thames, UK).

### 2.4. Quality Control Tests

#### 2.4.1. Cell Count

Cell counts were performed by automated methods (ABX MICROS 60, Horiba ABX for CBand Nucleocounter NC-100, Chemometec, Lillerød, Denmark, during the production process) according to validated procedures [24]. Viable and non-viable cell counts were determined by propidium iodide (PI, BD Biosciences, San Jose, CA, USA) staining on appropriately diluted MSC samples (0.2–0.75 × 10^6^ cells/mL).

#### 2.4.2. Microbiological Contamination

Sterility testing was performed using a previously validated method [24] by direct inoculation (European Pharmacopoeia, Eu. Ph. 2.6.27) and in both aerobic and anaerobic BacT/ALERT iFN culture bottles and incubated in the BacT/ALERT system (bioMérieux, Nürtingen, Germany). Finished product samples were also assayed for mycoplasma by culture methods (Eu. Ph.2.6.7). Endotoxin testing was performed with the limulus amebocyte lysate test (method D, gel clot, Ph. Eur. 2.6.14); the calculated limit for the product resulted 4.39 endotoxin units (EU)/mL, but specifications were set and validated at 0.25 EU/mL.

#### 2.4.3. Phenotyping and Viability (Flow Cytometry)

The MSC(CB) phenotype was determined by multicolor flow cytometry (FACSCanto II cytometer with Diva v.8.0, BD, Franklin Lakes, NJ 07417-1815, USA) following staining for surface molecules CD45 (Allophycocyanin(APC)-Cyanine(Cy)7-conjugated antibody, clone 2D1), CD73 (APC-conjugated antibody, clone AD2), CD90 (Phycoerythrin(PE)-Cy7-conjugated antibody, clone 5E10), and CD105 (Peridinin–Chlorophyll Protein (PerCP)-Cy5.5-conjugated antibody, clone 266) (antibodies from BD Biosciences, San Jose, CA, USA), using certified and validated protocols; all antibodies were from BD Biosciences, San Jose, CA, USA). The finished product was made by CD90- and CD105-positive and CD45-negative MSC(CB). Contaminant cells were identified as CD45-positive. Cell viability was assayed by flow cytometry of PI-stained white blood cells (0.5 × 10^6^ cells), following red blood cell lysis, or MSCs cells (0.1 × 10^6^) following staining for CD45 and CD90.

#### 2.4.4. Karyotyping

The genomic stability of MSC(CB) was monitored by our certified cytogenetics hospital-based laboratory using Q-banded chromosome karyotype analysis. Briefly, 50,000 MSC(CB)s were seeded in triplicate in Amnioflasks (EKAMF250, Euroclone spa, Pero, Italy) with either cell culture media or AmnioMAX™-II Complete Medium (ThermoFisher) and incubated for 48 h (humidified 37 °C, 5% CO_2_). At 30% confluence, colcemid was added (0.05 µg/mL), and cultures were incubated for 3 h. Metaphase spreads were prepared on microscope slides and treated with Quinacrine for Q-banding analysis, and chromosomal analysis followed official recommendations [27]. At least 20 metaphases were analyzed by fluorescence microscopy; digital images were captured by the Ikaros Karyotyping Platform (MetaSystems s.r.l., Milano, Italy).

#### 2.4.5. Adventitious Virus Analysis

Briefly, the cell pellet was resuspended in lysis buffer ATL (Qiagen, Hilden, Germany), and DNA and RNA were extracted using an automatic extractor EZ1 (Qiagen). Validated protocols were used to detect cytomegalovirus (CMV) and Epstein–Barr virus (EBV) by quantitative real-time methods (CMV ELITe MGB^®^ Kit, EBV ELITe MGB Kit; ELITechGroup, Torino, Italy) [28]. A qualitative RT-PCR method (Seeplex RV15 OneStep ACE Detection, Seegene, Seoul, Korea) was used to detect 15 respiratory viruses using a 7300 Real-Time PCR System (Applied Biosystems, Norwalk, CA, USA).

#### 2.4.6. HLA Typing

HLA-typing was performed using Sequence-Specific Oligonucleotide Probe Hybridization Assays (EFI standards, current edition).

#### 2.4.7. CFU-F

For CFU-F assays, 200 MSC(CB)s were seeded in a T25 flask and cultured for 12 ± 1 d before staining with gentian violet, as previously described [21].

#### 2.4.8. Fold Expansion

For FE calculations, 4000 MSC(CB)s/cm^2^ were cultured in a T75 flask for 7 d and counted following trypsinization.

### 2.5. Additional Tests

We performed additional characterization of MSC(CB) regarding extracellular matrix deposition, telomere length, and multi-lineage differentiation potential, with the aim to identify batch-to-batch variability.

#### 2.5.1. RNA Extraction and Real-Time qRT-PCR for Gene Expression

Total RNA was extracted from MSC(CB)s with the TRIzol reagent method (15596026; ThermoFisher), and total RNA was checked by spectrophotometry (ND-1000, NanoDrop Technologies, Wilmington, DE, USA). For the qRT-PCR assay, cDNA was synthesized from 500 ng of total RNA with SuperScript IV VILO (11756500; ThermoFisher). The resulting cDNA was diluted 1:10, and 1 µL was used as a template for a PowerUp SYBR Green Master Mix (A15780, ThermoFisher) reaction in a CFX96 thermal cycler (BioRad, Hercules, CA, USA). Relative gene expression levels were determined with a previously optimized ΔCt method, using the geometric means of endogenous *ACTB* and *GAPDH* mRNA levels [29]. Primer sequences are available upon request.

#### 2.5.2. DNA Extraction and Real-Time qPCR

For DNA extraction, cell pellets were lysed with 750 µL of room-temperature lysis buffer (20 mM TRIS (pH 7.44), 10 mM EDTA, and 100 mM NaCl) containing 50 µL of proteinase K (1 mg/mL) and 300 µL of 10% SDS. Samples were shaken overnight at 37 °C, extracted with 1 mL phenol/chloroform/isoamyl alcohol, and centrifuged (9000 rpm, 5 min). The aqueous phase was collected, and extraction/centrifugation was repeated; the washed aqueous phase was collected and combined with 0.1 volumes of 5 M NaCl and 1.5 mL of 100% EtOH and incubated at −80 °C for 30 min. Samples were then centrifuged at 4 °C (12,000 rpm, 15 min), and the supernatants were discarded, after which 1 mL of cold 70% EtOH was added to the pellet. Samples were vortexed and centrifuged at 4 °C (12,000 rpm, 5 min), after which the supernatants were discarded, and the pellets were resuspended in 50 µL of nuclease-free H_2_O. DNA concentrations were checked by spectrophotometry (Nanodrop, Nanodrop Technologies LLC, Wilmington, DE 19810, USA), and 10 ng of DNA was used as the template for real-time qPCR assays with PowerUp SYBR Green Master Mix in a CFX96 thermal cycler. Optimal amplification protocols for repeated telomeric sequences and single-copy reference gene *36B4* were as described previously [21]; telomere lengths were estimated with the ΔCt method, normalizing to the *36B4* signal. Primer sequences are available upon request.

### 2.6. ATMP Stability in the Storage Conditions

Stored batches were randomly checked on a rolling basis, testing three different certified batches every 2 years after stressing the storage conditions (“stress test”). The “stress test” consisted of repeatedly removing and replacing (for 30 s) the product in the nitrogen vapor three times within 3 min. Cells were then thawed in a water bath (37 °C) and combined with a 5% volume of HSA before testing identity, viability, purity, sterility, CFU-F, FE, and karyotype. Compliance criteria for stability were ≥90% CD90- and CD105-positive cells, CD45-negative cells; ≤2% CD45-positive cells; ≥80% PI-negative cells; no microbial growth; CFU-F ≥ 1; FE ≥ 2; and an absence of chromosomal abnormalities.

If at least two batches passed the test, the final product expiration date was fixed at the maximum storage time. For failed stability tests, the expiration date was fixed based on the most recent passed stability test.

### 2.7. ATMP Stability under Usage Conditions

Cryopreserved cell suspension was thawed in a water bath (37 °C, 8–10 min) or in a dry-thawing device (Barkey Plasmatherm, Leopoldshöhe, Germany) and then resuspended in a saline solution with 10% HSA and Anticoagulant Citrate Dextrose Solution, Solution A (ACD-A, Terumo BCT, Tokyo, Japan). Cells were tested for identity, clonogenic, and expansion ability after 30 min and for viability at 30, 120, and 270 min. The compliance criteria were as before.

### 2.8. Clinical Use of MSC(CB)s

In Europe, MSCs are classified as ATMPs [30,31], and Italian ATMP production plants must be authorized for medicinal product manufacturing by the national agency for drugs and can be also certified for therapeutic applications under a “hospital exemption” (HE) [32]. Our manufacturing plant is certified for both therapeutic and experimental applications after establishing ourselves within the regulatory framework for ATMP production. MSC(CB)s were used for the following purposes: (1) immunomodulation and (2) tissue repair. The clinical indications were Graft-versus-Host Disease (GvHD) and nephrotic syndrome (NS). One newborn affected by BPD was treated under HE and on an almost compassionate basis; therefore, it has not been considered for the scope of the present report. Patients with GvHD were treated under a “HE” application. For NS, we provided MSC(CB)s to pediatric patients in the context of two different clinical protocols: the first was a monocentric, prospective, open-label, single-arm phase I-II study in multi-drug-resistant idiopathic nephrotic syndrome, performed between April 2015 and April 2019 (Acronym KID’s01, EudraCT number 2011-001387-21) [33]; the second, still ongoing, is an open-label, single-arm, monocentric trial, with a rescue/second (adaptative) design addressed to maintain remission after immunosuppressive therapy withdrawal in steroid-dependent nephrotic syndrome (Acronym RACE, EudraCT number 2018-001162-42). To assess the clinical safety, the number of infusion-related adverse events was evaluated. Efficacy on GvHD was assessed as follows: complete remission (CR) was defined as the complete resolution of acute GvHD manifestations in all organs; partial remission (PR) as an improvement in GvHD stage in at least one of the involved sites without complete resolution and without worsening in any other organs. Regarding the clinical use of MSC(CB) in MDR-INS (KID’s 01 trial), the main efficacy outcomes was the response to therapy at 12 months, classified as partial remission if the urinary protein/urinary creatinine ratio (uPr/uCr) was between 0.2 and 2 mg/mg or complete remission if uPr/uCr was <0.2 mg/mg. Patients were categorized as “responders” if they achieved a partial or complete remission; otherwise, they were considered “non-responders”. The reduction of ongoing immunosuppressive and antiproteinuric agents was a secondary efficacy endpoint. In the second other trial in SDNS, the main objective was to evaluate whether CF-CB-MSC therapy is able to prevent NS recurrence for at least 6 months after complete withdrawal of immunosuppressive treatment in children with SDNS.

## 3. Results

### 3.1. Tissue Procurement

Three CB units were received from our bank and used for GMP-level MCB production; the characteristics of these available batches are reported in Table 1. Interestingly, all CB units contained <10% monocytes in the nucleated cell fraction; in addition, a previous study found that their volume was <90 mL in two-thirds of cases [21].

### 3.2. GMP Facility, MSC(CB) Manufacturing Process and Quality Controls

There were no deviations in the Heating–Ventilation–Air-Conditioning (HVAC) system’s functioning and controls. Media fills were performed twice per year, as described by [24], and they were always compliant.

**Figure 2:**
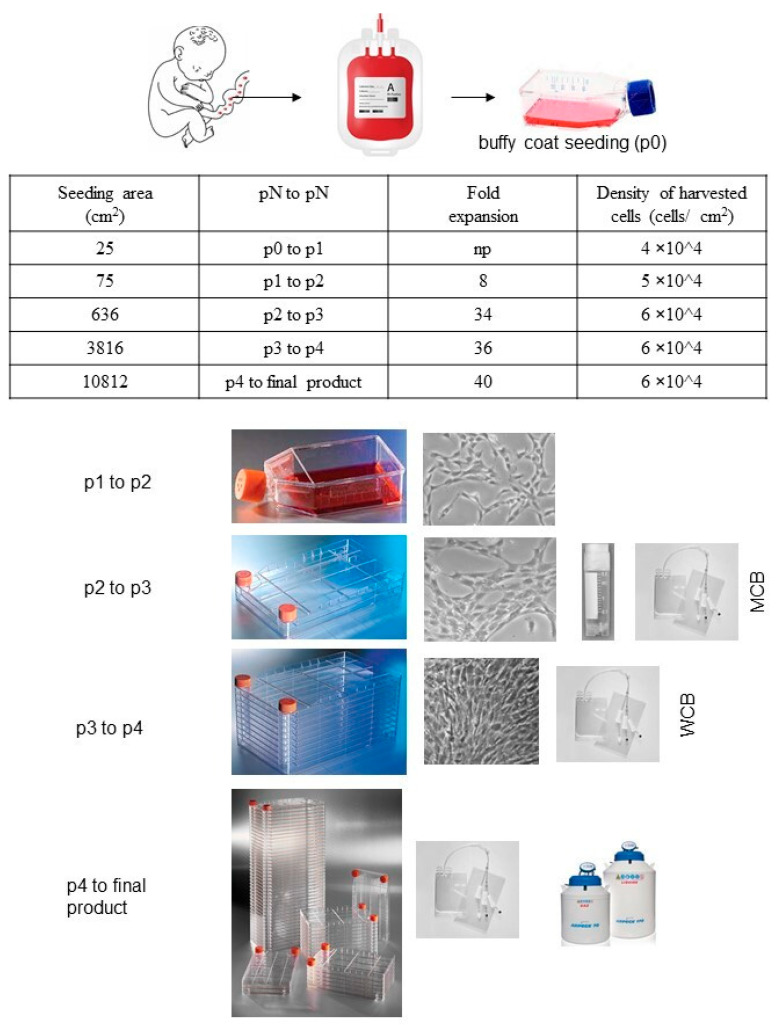
Scale of cell production along the manufacturing process. P, passage; MCB, Master Cells Bank; WCB, Working Cells Bank.

Approved reagents are listed in Table 2. Regarding MSC manufacturing process, from three CB donations, we obtained an MCB made of 1.29 × 10^8^ MSCs. We observed a mean of 5 FE in the P3-P4 expansion and 21 FE in the P4-P5 expansion, giving total WCB and final product yields of 1.58 × 10^9^ (±1.79 × 10^9^) and 7.14 × 10^10^ (±8.37 × 10^10^), respectively (see Figure 2). Thus, a WCB containing 4.74 × 10^9^ MSC(CB) can be produced (with a mean P3-P4 FE of 39.4), and the yield of final product obtained from three single CB donation totals, 2.14 × 10^11^ MSCs, for clinical use. This yield makes it possible to treat large patient cohorts of > 500 subjects (3–4 doses at 1.5–2 × 10^6^ MSC/kg for an average 70 kg patient) under the hospital-exemption procedure and also supports large phase I/II/III clinical trials. All batches of final product tested negative for endotoxins, bacteria, mycoplasma, and adventitious viruses and were characterized for GMP compliance prior to release (see Table 3). Because the finished product is a cryopreserved cell suspension that requires thawing, we assessed cell viability and phenotype 30 min after thawing.

### 3.3. Additional Tests

**Figure 3:**
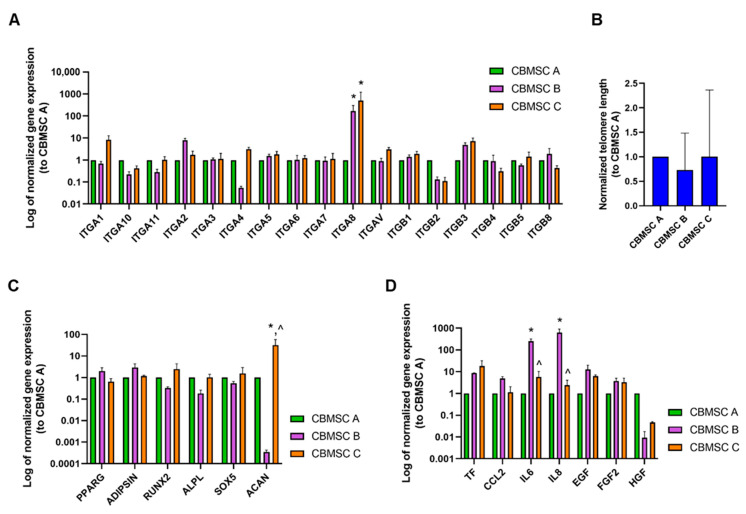
For information only characterization of cord blood-derived mesenchymal stromal cells. (**A**) Histograms show gene expression of integrins normalized to CBMSC A; data are presented as mean ± standard deviation (SD) (n = 3). Statistical analysis was by two-way ANOVA followed by Tukey’s multiple comparisons post-hoc test; * *p* < 0.05 vs. CBMSC A. (**B**) Histograms show telomere length normalized to CBMSC A; data are presented as mean ± SD (n = 3). Statistical analysis was by non-parametric Kruskal-Wallis test followed by Dunn’s multiple comparisons post-hoc test. (**C**) Histograms show transcript levels of differentiation-associated genes normalized to CBMSC A; data are presented as mean ± SD (n = 3). Statistical analysis was by two-way ANOVA followed by Tukey’s multiple comparisons post-hoc test; * *p* <0.05 vs. CBMSC A, ^ *p* < 0.05 vs. CBMSC B. (**D**) Histograms show gene expression of secreted proteins normalized to CBMSC A; data are presented as mean ± SD (n = 3). Statistical analysis was by two-way ANOVA followed by Tukey’s multiple comparisons post-hoc test; * *p* < 0.05 vs. CBMSC A, ^ *p* < 0.05 vs. CBMSC B. Abbreviation list: CBMSC, cord blood mesenchymal stromal cells; SD, standard abbreviation.

Three MSC(CB) batches were characterized in triplicate. We found no significant batch-to-batch variability regarding gene expression of integrin monomers (with the exception of ITGA8, Figure 3A), telomere stability and erosion (Figure 3B), or transcription levels of proteins relevant to differentiation toward adipocytes (i.e., *PPARG* and *ADIPSIN*), osteogenesis (i.e., *RUNX2* and *ALPL*), and chondrogenesis (i.e., *SOX5* and *ACAN*) (Figure 3C). The expression of *ACAN* did vary between batches, and given its structural role in the cartilage extracellular matrix, its expression could influence chondrocyte differentiation. Two genes coding for inflammatory cytokines (*IL6* and *IL8*) were significantly more expressed in one MSC batch (Figure 3D).

### 3.4. Stability

Final product stability was characterized for 27 samples (11 batches) for cryopreservation durations of 3–76 months and found to be suitable regarding identity (96.8 ± 3.2%), purity (99.4 ± 0.6%), and viability (84.3 ± 10.6%). All thawed products were sterile with normal karyotypes and expanded in culture (FE 8.0 ± 4.8, n = 27) to produce the expected CFU-F (17.9 ± 17.4, n = 26). Only 1 of the 12 batches stored for > 60 months passed quality control measures; therefore, the expiration of MSC final products is fixed at 56 months of storage.

Our stability study of cell viability after thawing (Supplementary Table S2) revealed that the percentage of PI-negative cells remained stable for 30 min after thawing and then declined below 80%; during this time, cells also maintained their identity and proliferation potential.

### 3.5. Clinical Use

From March 2012 to June 2023, 263 of our GMP-certified MSC(CB) preparations were administered as ATMPs in 40 subjects (18 female) affected by GvHD (n = 8), nephrotic syndrome (NS, n = 31), or bronco-pulmonary dysplasia of the newborn (n = 1) in approved clinical trials (n = 31) or for therapeutic use following single-patient authorization (n = 12; eight with GvHD, three with NS, and one with BPD). One subject with NS was also treated after being treated in the KID’s01 study, on a “single-use approval”, for disease recurrence. Most pediatric patients (age < 18 years) were affected by NS (78.5%), while most adults (eight of nine) were affected by GvHD. Cells were administered through intravenous injection via the peripheral or central vein; in only one case of BPD was endo-tracheal administration used. Each patient received the prescribed dose divided into 3–12 preparations (median 6) over 2–4 administrations (median 3), and each infusion contained 19–142 × 10^6^ MSCs (median 70 × 10^6^). The most used schedule was based on three doses at a 1–2-week interval, while a discretionary fourth dose was given if required by the experimental protocol as a consolidation dose (n = 7) or as a rescue therapy (N = 1). Details of patient demographics and experiments are shown in Table 4. No infusion-related adverse events have been reported in any of the treated patients [33].

For GvHD, the results are reported in Table 5. Data are available until 18 months from MSC(CB) infusion. A CR was registered in two out of the eight treated patients (one affected by NHL and the other one by MM, both with gut involvement). One of the two patients with CR (patient #7 in Table 5) died after 3.5 months due to thrombotic thrombocytopenic purpura. Two patients did not respond: one of them (patient #5 in Table 5) showed graft failure and leukemia relapse; the disease was poorly controlled with standard chemotherapy, and he died 10 months after MSC infusion. The two patients with overlap GvHD showed both a PR with stable clinical conditions at the last follow-up visit.

## 4. Discussion

Many unmet clinical needs have the potential to be addressed because of recent discoveries and advances in stem cell regenerative potential, gene-editing tools, and clinical transplantation methods [34,35,36]. In addition to other actors, multilineage stem/stromal cells may play a critical role [37], and their potential as immunomodulatory and anti-apoptotic agents has inspired clinical researchers to innovate. In our experience, MSC(CB) has proven to be safe and to exert beneficial effects on patients affected by GvHD, the majority of which reached complete or partial remission. Indeed, interesting information came up from the KID’s01 study, in which a subgroup of pediatric patients with MDR-INS resistant to a median of three previous lines of therapy with a lower baseline uPr/uCr achieved partial or complete remission. These encouraging results prompted us to go further with a phase II study (the RACE trial) in which the immunosuppressive effect of MSC(CB) has been tested in steroid-dependent patients, with the aim to free them from heavy pharmacologic treatment. Notably, none of the patients of the KID’s 01 study showed the development of donor-specific HLA antibodies (DSAs). This was essentially in contrast with previously reported studies with (BM) and (AT)MSC. The lack of DSA after (CB)MSC might be correlated to their immunological naivety but deserves additional investigation supported by a larger patient cohort. On the other side, also the occurrence of autoimmunity never occurred in our experience with the patients treated with (CB)MSC, thus confirming their immunological safety. The main candidate indication of MSC(CB) may be therefore as an immunosuppressant agent both in refractory severe immune-mediated and in steroid-dependent diseases [38]. These clinical findings have, as biological counterparts, the peculiar trophic, anti-inflammatory, and anti-apoptotic properties of MSC(CB), as we and others previously documented at the RNA, protein, and functional levels. In particular, the differentiation-associated genes (see Figure 3, panel C) have been already validated as molecular counterparts of the in vitro differentiation capacity of (CB)MSC in our previous works [16,21,39]. Nevertheless, it is still not clear which are the mechanisms of action underlying these effects. In particular, MSCs are not detectable a few hours after systemic administration, and they are trapped in the lungs, where they release anti-inflammatory proteins [40]. Specifically, following the contact with recipient cytotoxic T-lymphocytes, MSCs trigger apoptosis in vivo and are phagocyted by monocytes [41]. It has already been proved in murine GvHD animal models that MSC phagocytosis increases IDO expression [42] with beneficial consequences on immunomodulation. Based on this putative mechanism of action and starting from these precious clinical results, larger clinical trials on a well-defined patient population will give more precise insights into the clinical appropriateness of this promising ATMP. Despite the very encouraging clinical results that we reported, the potential of MSC(CB) has not yet been fully exploited, and few similar manufacturing experiences are part of the clinical arena. In addition to other factors that occurred at the beginning of our work (e.g., regulatory agency and clinicians’ unfamiliarity with these new drugs), technical aspects also hampered the wider use of MSC(CB). Indeed, the large-scale manufacturing of CB-derived ATMPs is extremely challenging because of the large volumes of certified high-quality starting materials required for high-yield medicinal products [21,43]. Here, we designed an MSC(CB) manufacturing process that maximizes expansion potential with a cell-banking approach that has several advantages: (1) each production lot derives from a well-characterized common starting source; (2) an MCB/WCB system allows for storage of intermediate products that can be expanded without requiring new starting material collection and isolation, thus reducing space and cost requirements; and (3) WCB expansions can be scheduled based on the balance of supply and clinical demand. Though CB banking for hematopoietic cell transplantation is well established, we found no comparable reports on CB banking for MSCs and few on MSCs derived from umbilical cord tissue (another neonatal source of MSCs) [44,45,46,47] that may serve as a best comparator with our experience. In this regard, Oliver-Vila and colleagues designed an MSC manufacturing process starting from relatively few samples (e.g., two umbilical cords), as we did, and established an MCB/WCB system with a production yield similar to ours [44]. This group used a different methodology and still observed an excellent senescence profile for their neonatal-derived MSCs, though they did not report on clinical applications and administration schedules for the final product. We always strive to find and exploit the therapeutic potential of CB while considering the most updated scientific and technical information. Cord blood provides the youngest cells that can be collected non-invasively and remain viable in storage for several decades [48,49]. A young MSC source is critical for regenerative medicine because increased age is associated with an organism-wide increase in the expression of secreted senescence factors that impair tissue function [50,51]. Although cells from older patients can be reprogrammed, the process is not efficient and generates cells with genomic damage. Therefore, younger tissues are ideal for obtaining iPSCs, extracellular vesicles, mitochondria, and MSCs, mitigating the risks of age-related cellular changes [52,53]. All starting materials for GMP cell banking must be stable and expandable, and CB-derived MSCs meet these requirements. The isolation of MSC precursors from CB does not require extensive manipulation, while protocols for isolation from umbilical cord tissue require pre-isolation treatments and/or cryopreservation that may affect the final expansion results [45]. Also, the choice to use FBS instead of animal-free alternatives has been proven to be successful due to its consistency, regulatory acceptance, reliability, cost-effectiveness, and strong track record in clinical trials [54]. In summary, with a well-defined manufacturing process, we were able to generate sufficient inventory for the treatment of large cohorts of patients [55,56,57,58,59].

Beyond stringent quality-control testing, we also investigated the specific properties of each MSC(CB) batch. We found that MSC(CB) integrin expression is critical for MSC homing and survival signaling and may provide a cell-type-specific profile [60,61]. In particular, ITGA8 is involved in mesenchymal–epithelial cell connection and extracellular matrix deposition [62], and it may influence the wound-healing potential of MSCs. We also found batch variability in the expression of IL6 and IL8 that may be relevant to acute and chronic inflammation, which may modulate tissue-remodeling processes such as angiogenesis [55]. These wound-healing and anti-inflammatory properties are typical of MSCs from neonatal sources and distinguish them from adult tissue-derived MSCs [56]. Though MSC(CB) batches were largely similar, detailed knowledge of molecular differences may be useful in achieving the desired therapeutic effect in a clinical setting. Our studies of the functional heterogeneity of MSC(CB) confirm the findings of others studying neonatal-derived MSCs and support their use in personalized medicine [57,58].

## 5. Conclusions

We conclude that an accurate approach to GMP process development, an extensive characterization of the final product, and a precise definition of the clinical study design are critical factors for success in ATMP pharmaceutical development and therapeutic applications.

## Figures and Tables

**Table 1:** Cord blood unit: donation and donor characteristics. Low cellularity means cell count <1.5 × 10^9^ total nucleated cells. CS, caesarean section; ND, natural delivery.

Starting Material ID	#1	#2	#3
Banking exclusion criteria	Low cellularity	Low volume	Low volume
Volume (mL)	91.5	59.5	51.3
Total nucleated cells (×10^3^/µL)	10.5	8.5	9.8
% Monocyte	4.3	6.2	6.3
Gestational age (weeks)	40	41	40
1st minute Apgar score	10/10	9/10	9/10
5th minute Apgar score	10/10	10/10	10/10
Type of delivery	CS	ND	CS
Child gender	M	F	F
Child weight at birth (kg)	3.40	2.96	3.28

**Table 2:** Cord blood-derived mesenchymal stromal cell manufacturing: selection and qualification of the key reagents.

Product	Supplier	Reference	Specification
Human albumin 200 g/L	Kedrion, Lucca, Italy	A.I.C. 022515163	Blood product for human use (any Marketing Authorization Holder—MAH).
ACD-A	Terumo BTC, Tokyo, Japan	40804	Class 2 medical device manufactured according to Directive 93/42/EEC.
Citrate phosphate dextrose CPD	SALF SpA Bergamo, Italy	A.I.C. 031328	Medicinal product for human use.
DMSO	Cryoserv—Mylan Institutional Canonsburg, PA, USA	67457-178-50	Manufactured according with GMP, sterile and with endotoxin level < 0.5 EU/mL.
FBS Australian	ThermoFisher Scientific Thermo Fisher Waltham, MA, USA	10101-145	EDQM-certified of suitability gamma irradiated sterile serum with an endotoxin level < 0.5 EU/mL.
PBS	MacoPharma, Tourcoing, France	0120020	Xeno-free solution tampon, sterile and with endotoxin level < 0.5 IU/mL.
Physiological Solution NaCl 0.9%	Baxter, Milano, Italy	A.I.C. 035715010	Medicinal product for human use e.v.
TrypLE Select	Thermo Fisher Scientific Thermo Fisher Waltham, MA, USA	12563-011	A sterile free of any animal origin element trypsin with endotoxin level < 1.0 EU/mL.
αMEM 675 mL	MacoPharma, Tourcoing, France	0110020	A sterile xeno-free medium with endotoxin level < 0.5 UI/mL.

**Table 3:** Cord blood-derived mesenchymal stromal cell manufacturing: release tests and quality controls performed on the finished product batches (N = 19). Numerical data are reported as mean (±SD). PI, propidium iodide; SIGU, Società Italiana di Genetica Umana; n.d., not detected; EFI, European Federation of Immunogenetics.

	Test Name	Method	U.M.	Result	Specifications
Release test	Purity	Flow cytometry (Ph. Eu. 2.7.24)	CD45− CD90+ CD105+ cells (%) ^§^	97.3 ± 2.7	≥90
	Contaminants		CD45+ cells (%) ^§^	0.6 ± 0.5	≤2
	Viability		PI- cells (%)	92.7 ± 4.6	≥80
	Sterility	Ph. Eu. 2.6.27	/	Sterile	Sterile
	Bacterial endotoxin testing	Ph. Eu. 2.6.14	EU/mL	0.245 ± 0.0	<0.25
	Mycoplasma	Ph. Eu. 2.6.7	/	No growth	No growth
	Karyotype	Q-banding (SIGU guidelines)	/	46,XX/46,XY	46,XX/46,XY
Additional test *	Extended immunophenotype	Flow cytometry (Ph. Eu. 2.7.24)	CD45− CD73+ CD105+ cells (%)	96.5 ± 4.0	none
			CD45− CD73+ CD90+ cells (%)	95.8 ± 5.3	none
	Viability (restricted)	Flow cytometry (Ph. Eu. 2.7.24)	CD45− CD90+ PI- cells (%)	91.8 ± 5.2	none
	Post-thawing viability	Flow cytometry (Ph. Eu. 2.7.24)	PI- cells (%)	±	≥80
	Adventitious viruses	PCR (Ph. Eu. 2.6.21)	CMV-DNA	n.d.	n.d.
			EBV-DNA	n.d.	n.d.
			Respiratory viruses RNA/DNA	n.d.	n.d.
	HLA typing	Sequence-Specific Oligonucleotide Probe (SSOP) Hybridization Assays (EFI standards, current edition)	HLA-A-B-DR donor	Correspondence to the donor HLA	Correspondence to the donor HLA

* This term identifies tests that are performed not to release the product but for statistical or research purposes. ^§^ % of viable cells.

**Table 4:** Demographic data of the subjects treated with cord blood-derived mesenchymal stromal cells (N = 40). The parameters marked with * are reported as median (range). GvHD, Graft-versus-Host Disease; NS, nephrotic syndrome; BPD, bronchopulmonary dysplasia.

Demographics	
Female gender (N; %)	18; 42.8
Age (years) *	12 (0.5–60)
Subjects < 18 years of age (N; %)	33; 78.5
Weight (kg) *	42 (2.7–83.6)
Subjects ≥ 18 years of age *	57.1 (41.2–80)
Subjects < 18 years of age *	34.7 (2.7–83.6)
Disease (n of total subject; n of subjects > 18 years of age)	
GvHD	8; 8
NS	33; 1
BPD	1; 0
Treatment	
Cell dose (×10^6^)/KG *	1.6 (1–10)
Cell dose (×10^6^)/kg in subjects ≥ 18 years of age *	1.7 (1.4–3)
Intravenous administration (n)	41
Subjects who received 3 administrations (n; %)	30; 71.4
Interval between treatments (days) *	8 (3–42)

**Table 5:** Characteristics and clinical outcome of patients affected by Graft-versus-Host Disease (GvHD). AML, acute myeloid leukemia; ATG, anti-thymocyte globulin; CyA, cyclosporine A; CR, complete remission; D, deceased; ECP, extracorporeal photopheresis; MM, multiple myeloma; MMF, mycophenolate mofetil; NHL, non-Hodgkin lymphoma; OS, overlap syndrome; PR, partial remission; NR, no response.

Patient No.	Age	Diagnosis	GVHD Grading, Type (and Site)	Treatment before MSC(CB) Infusion	Infusions (No.)	Response (Site of Response)	Treatment after MSC(CB) Infusion	State at 18-Month Follow-Up
1	52	NHL	Grade IV aGvHD (gut)	Steroid + MMF + ECP	2	CR	ECP suspension; steroid reduction	Cutaneous cGVHD
2	49	NHL	Grade IV aGvHD (skin; gut)	Steroid + MMF + ECP	3	PR (limited to gut)	MMF reduction	OS
3	22	MDS	Grade IV aGvHD (skin; gut)	Steroid	4	PR (limited to skin)	none	D (1)
4	51	AML	OS	CyA + steroid + ECP	3	PR (limited to skin)	CyA + steroid + ECP	D (3.5)
5	20	AML	IV (gut)	Steroid; Rituximab; Etanercept; ATG	2	NR	None	D (10)
6	22	MDS	IV (skin; gut)	Steroid	3	NR	None	D (1)
7	50	MM	IV (gut)	Steroid + MMF + ECP	3	CR	ECP suspension; steroid and MMF reduction	D (3.5)
8	59	AML	OS	CyA + steroid + ECP	3	PR (limited to skin)	CyA + steroid +ECP	OS

In the case of deceased patients, the duration of survival after mesenchymal stromal cell infusion is expressed in months (in brackets).

## Data Availability

Data are available upon request.

## Supplementary Materials

The following supporting information can be downloaded at: https://www.mdpi.com/article/10.3390/cells13121066/s1, Table S1: Cord blood unit: starting material specifications. CB: Cord Blood; CPD: Citrate-phosphate-dextrose; TE: Tissue Establishment; SM: Starting Material.; Table S2: Cord blood-derived mesenchymal stromal cells: stability in the usage conditions. Time 0 indicates pre-thawing samples.

## Abbreviations

ACD-A	Anticoagulant Citrate Dextrose Solution, Solution A
AIFA	Agenzia Italiana del Farmaco—Italian Drug Agency
ATMP	Advanced Therapy Medicinal Product
BM	bone marrow
CB	cord blood
CMV	cytomegalovirus
DMSO	dimethyl sulfoxide
EBV	Epstein–Barr virus
EU	endotoxin units
FBS	fetal bovine serum
FE	fold expansion
GMP	Good Manufacturing Practice
HAS	human serum albumin
LL	long-living
MCB	master cell bank
MSC	mesenchymal stromal cell
PI	propidium iodide
SL	short-living
WBC	working cell bank
