# Supplementary material for: Off-the-Shelf Cord-Blood Mesenchymal Stromal Cells: Production, Quality Control, and Clinical Use
Source: Cells. 2024 Jun 19;13(12):1066. doi: 10.3390/cells13121066 (PMC11202005; doi:10.3390/cells13121066)
Supplement: Supplementary file 1 [file cells-13-01066-s001.zip › cells-3036376-supplementary.pdf]

Table S1. Cord blood unit: starting material specifications. CPD: Citrate-phosphate-dextrose; CB: Cord Blood; TE: Tissue Establishment; SM: Starting Material.

**DEFINITION**	Tissue:<br>- Umbilical cord blood collected from the placenta and umbilical cord vessels after full term delivery
**ORIGIN**	Human:<br>- Healthy donors (see Table 2 for details)
**METHOD OF COLLECTION AND INITIAL HANDLING**	- "In utero" collection after vaginal delivery or "ex-utero" collection after cesarean section<br>- Closed collection system (bags) with anticoagulant (CPD)<br>- No other manipulation or sampling before being received by the TE
**TIME BETWEEN COLLECTION AND ARRIVAL AT THE GMP MANUFACTURING PLANT**	- ≤ 24 hours
**CARACTHERISTICS OF THE DONATION AND OF THE DONOR**	- Voluntary donation, after signing informed consent form by the mother of the newborn<br>- Health donor: absence of infectious or genetic diseases as defined by the exclusion criteria for CB donation in the pertinent Italian national laws (Law Oct 21st 2005, n. 219, Decree 2.11.2015) as per anamnestic information from the mother, the father and other available relatives as requested by the current anamnestic questionnaire of the TE at the time of collection
**ADDITIONAL CARACTHERISTICS AVAILABLE IN ONE WEEK FROM COLLECTION**	- Analysis performed by the TE on the "donation sample" (that is the peripheral blood sample collected by the mother the day of the delivery) tested negative: HBsAg, antibodies anti-HCV, anti-HIV 1-2, anti-HTLV I-II, HCV/HIV1 RNA - HBV DNA (NAT), anti-Treponema Pallidum
**1st LEVEL SPECIFICATIONS**<br>(Available at the receipt of the SM; only if they are conform the SM can be introduced in the manufacturing process)	- TE declaration on the availability and conformity of the medical history and informed consent from the mother<br>- CB donation labeled as requested by the JACIE-FACT current standard and the Italian national law (Law Oct 21st 2005, n. 219, DLvo 2.11.2015) with the Single European Code (SEC)<br>- CB container intact
**2nd LEVEL SPECIFICATIONS**<br>(Available only after that the SM has been introduced in the manufacturing process; if they are not compliant, the manufacturing process is stopped and/or the final product cannot be released)	- Analysis performed by the TE on the "donation sample" (that is the peripheral blood sample collected by the mother the day of the delivery) tested negative: HBsAg, anti-HCV, anti-HIV 1-2, anti-HTLV I-II, HCV/HIV1 RNA - HBV DNA (NAT), anti-Treponema Pallidum<br>- Sterility testing: negative (performer by the GMP manufacturing facility)
**STORAGE**	- During the time requested by the 1st level controls: 4-8°C
**VOLUME**	- Undefined: all the CB units can be introduced into the manufacturing process if compliant to 1st level specifications (see above)

Table S2. MSC(CB) stability in the usage conditions. Time 0 indicates pre-thawing samples.

SAMPLE	CRYO-PRESERVATION TIME (MONTHS)	POST-THAWING TIME (MINUTES)	VIABILITY (% CELLS PI-)	IDENTITY (% CELLS CD45-CD90+CD105+)	CFU-F	FOLD EXPANSION
#1	24	0	95	98.7	23	20
		30	82	97.7	21	17
		120	76			
		270	68			
#2	12	0	96	91.6	10	35
		30	82	91.9	9	47
		120	75			
		270	68			
#3	2	0	95	94.1	15	11
		30	90	93.8	17	11
		120	81			
		270	78			
Mean ± standard deviation		0	95 ± 1	95 ± 4	16 ± 7	22 ± 12
		30	85 ± 5	94 ± 3	16 ± 6	25 ± 19
		120	77 ± 3			
		270	71 ± 6			
